# Elevated androstenedione in young adult but not early adolescent prenatally androgenized female rats

**DOI:** 10.1371/journal.pone.0196862

**Published:** 2018-05-03

**Authors:** Ami B. Shah, Isaac Nivar, Diana L. Speelman

**Affiliations:** Department of Biochemistry, Lake Erie College of Osteopathic Medicine, Erie, PA, United States of America; University of Missouri Columbia, UNITED STATES

## Abstract

**Background:**

Elevated testosterone (T) is routinely reported as a marker of hyperandrogenemia in rodent models for polycystic ovary syndrome (PCOS). In women with PCOS, elevated serum androstenedione (A4) is associated with more severe phenotypes, including a positive correlation with serum T, DHEAS, free androgen index (FAI), LH, and LH/FSH ratio. Furthermore, A4, along with calculated free T and FAI, was identified as one of the best predictors of PCOS in adult women of all ages (18 to > 50 y).

**Objective:**

The objective of this study was to investigate serum A4 levels in early adolescent and young adult prenatally androgenized (PNA) female rats, a model for PCOS.

**Methods:**

Pregnant rats were injected with 5 mg T daily during gestational days 16–19 (PNA rats, experimental group) or an equal volume of vehicle (control group). Female offspring of both groups had tail vein blood drawn for serum analysis at 8 and 16 weeks of age. ELISAs were used to quantify serum A4 and T levels.

**Results:**

Serum A4 and T were elevated in 16-week-old PNA rats compared to controls. There was no significant difference in either hormone at 8 weeks of age.

**Conclusions:**

The PNA rats demonstrated elevated serum A4 and T in young adulthood, as has been observed in women with PCOS, further validating this as a model for PCOS and underscoring the importance of serum A4 elevation as a parameter inherent to PCOS and a rodent model for the disorder. Significant A4 elevation develops between early adolescence and early adulthood in this PNA rat model.

## Introduction

Polycystic ovary syndrome (PCOS) is the most common endocrine and metabolic disorder in women, affecting 5 to 15% of women of reproductive age [1, 2]. The syndrome may manifest itself as early as the second decade of life [3, 4], although it may not be diagnosed until the third decade when the reproductive features of the disorder are more apparent [5–7]. PCOS can impact female endocrine, reproductive, metabolic, and psychological health from puberty to menopause and beyond [8–11]. Common findings include menstrual irregularity secondary to oligo-ovulation, subfertility, hirsutism, acne, weight gain, and anxiety [1, 12]. Women with PCOS often exhibit glucose intolerance and insulin resistance, and are much more likely to develop type 2 diabetes [13, 14]. Despite its prevalence and impact on female health, the cause of the disorder remains unknown.

The 2003 Rotterdam criteria are often used to make a PCOS diagnosis, which requires the presence of at least two of the three features of hyperandrogenism (clinical or biochemical), oligo- and/or anovulation, and polycystic ovaries, as well as the exclusion of other endocrine disorders as a cause of these features [15]. The diagnostic criteria proposed both by the 1990 NIH Consensus Conference recommendations and by the 2009 Androgen Excess Society Task Force report require the presence of hyperandrogenism in order to make a diagnosis of PCOS [16, 17]. Interestingly, a recent study using steroid profiling in a study of PCOS and control women indicated that 90% of patients in the PCOS population had hyperandrogenism, including women originally classified as having oligo-amenorrhea and polycystic ovary morphology without clinical signs of hyperandrogenism [18]. These findings further underscore the importance of biochemical hyperandrogenemia in this syndrome, both for diagnosis and likely for a central role in its pathogenesis.

There is strong evidence from animal models and human studies to support that androgen excess contributes to the development and exacerbation of the presentation of this disorder, including the development of metabolic dysfunction. Prenatal or postnatal exposure to androgens results in most of the features of PCOS in rodent, sheep, and non-human primate models, including the presence of hyperandrogenemia, oligo- or anovulation and disrupted reproductive cycling, subfertility, elevated LH and altered LH pulsatility, obesity, dyslipidemia, fatty liver, glucose intolerance and insulin resistance, and altered adipokine secretion [19–36]. In women with PCOS, hyperandrogenism is a consistent feature and important diagnostic criterion, with the extent of biochemical hyperandrogenism often correlating with the severity of the disorder [37–39]. This hyperandrogenism primarily reflects an elevation in the androgens of ovarian origin, including testosterone (T), androstenedione (A4), and dehydroepiandrosterone (DHEA), although adrenal DHEAS may also be elevated [40–42].

A4 is a weak androgen and precursor for T synthesis, with most conversion occurring in the ovary, but some in the adipose and peripheral tissues as well [43, 44]. A4 was recently identified as one of the best predictors for having PCOS, along with calculated free testosterone and free androgen index, in women 18–44 years old [45]. While biochemical analysis of hyperandrogenism often utilizes T, it is important to note that A4 may be elevated in women with hirsutism and PCOS even when T levels are normal [46–48]. Furthermore, O'Reilly and colleagues have demonstrated that patients with elevated A4 and normal T have nearly equivalent risk for metabolic disease as those with elevated T alone [47], underscoring the importance of measuring this androgen when evaluating women for PCOS. However, others have reported that A4 elevation may be slightly attenuated by obesity and that a higher A4/free T (fT) ratio correlated with an improved metabolic profile [48]. While elevated A4 levels are reported to correlate well with PCOS status and severity in women [37], it is not known whether A4 elevation is present around the time of puberty. Similarly, while some have reported elevated A4 levels in rodent models for PCOS [49, 50], it is unknown whether this elevation is present during early adolescence or only in adulthood in these models. Using a prenatally androgenized (PNA) rat model for PCOS, we investigated if there is a significant elevation in A4 in the PNA rats, both at 8 weeks (early adolescence) and 16 weeks of age (young adulthood).

## Materials and methods

### Materials

Testosterone, sesame oil, benzyl benzoate, and Giemsa stain were purchased from Sigma Aldrich (St. Louis, MO, USA). Testosterone ELISA kits (item no. 582701) were purchased from Cayman Chemicals (Ann Arbor, MI, USA) and androstenedione ELISA kits (item no. ab108672) were purchased from Abcam (Cambridge, MA, USA).

### Animals

All procedures involving rats were approved by the Lake Erie College of Osteopathic Medicine Institutional Animal Care and Use Committee (approved protocol #16–03). Sprague-Dawley rats were given ad libitum access to water and standard rat chow (Hilltop Lab Animals, Inc.; Scottdale, PA, USA). Rats were housed in a temperature-controlled animal facility (23–25°C) with a 12-hour light/dark cycle.

### Prenatal androgenization of rats

Each breeding pair was housed together in a mating cage until the presence of a vaginal plug confirmed pregnancy (gestational day 1, GD1). The male and female breeders were then housed separately. On the mornings of gestational days 16–19, the pregnant dams were subcutaneously injected with 5mg testosterone dissolved in 500 μL of a 4:1 sesame oil-benzyl benzoate mixture or 500 μL of the vehicle alone. Pups were weaned and separated by sex at 3 weeks of age, and housed 2–3 rats per cage.

### Vaginal cytology

In the days leading up to the tail vein blood draw, a daily vaginal smear was used to monitor the rat estrous cycle. A moist cotton swab was used to collect vaginal epithelial cells, which were then transferred to a slide and stained with Giemsa for 2 minutes at room temperature followed by a water rinse for 2 minutes. Vaginal smear samples were then imaged at 20X magnification on an inverted light microscope. Estrous phase determinations were made as described by others [51, 52]. Blood draws were conducted on the morning of estrous whenever vaginal smear was possible (no vaginal fusion present) in order to minimize any potential effects of cycling hormones on the parameters measured [53, 54].

### Tail vein blood draw and serum collection

At 8 weeks and 16 weeks of age, rats were anesthetized with 80 mg/kg thiobutabarbital in normal saline prior to collection of a 1 mL tail vein blood sample with a 25G butterfly needle and syringe. A drop of blood was used to determine serum glucose levels using a standard glucometer (OneTouch Ultra2; LifeScan, Inc., Wayne, PA, USA). Blood samples were allowed to coagulate for 10–20 minutes on ice, then separated by centrifugation at 2,000 rpm for 5–10 minutes, followed by transfer of the serum to a clean microfuge tube on ice. Serum samples were stored at −80°C until thawed on ice for analysis by ELISA.

### Tissue collection

At 16 weeks of age, rats were anesthetized with 200 mg/kg thiobutabarbital in normal saline. Once the animals were unresponsive to toe-pinch, they were transcardially perfused through the ascending aorta with normal saline. Ovaries and uterus were carefully dissected from each animal and weighed.

### Histological examination of ovaries

Following dissection at 16 weeks of age, ovaries were fixed in 10% formalin for 2 days, and then processed by standard histological protocol and embedded in paraffin. The ovaries were serially sectioned at 8 μm thickness, mounted on a glass slide, then deparaffinized in xylene and hydrated through an ethanol series (100, 90, 80, 70, and 50%) and water. Sections were stained with hematoxylin and eosin prior to mounting a glass coverslip. Ovarian sections were observed under a light microscope at 4x and 10x magnification.

### ELISAs

Serum samples from 16 8-week-old rats (8 control rats from 3 litters and 8 PNA rats from 3 litters) and 16 16-week old rats (8 control rats from 3 litters and 8 PNA rats from 3 litters) were used for analysis by ELISA. Undiluted serum samples were analyzed in duplicate within a single assay. ELISA analyses were conducted and data analyzed according to the manufacturers' protocols. For the serum samples analyzed by an ELISA for testosterone, absorbance values at 420 nm were determined. For the same samples analyzed by an ELISA for androstenedione, absorbance values at 450 nm were determined. Intra-assay coefficients of variation were less than 10%.

### Statistical analysis

GraphPad Prism 6 software (San Diego, CA, USA) was used for statistical analysis. All data were normally distributed for each group. Two-way ANOVA with Tukey's multiple comparisons were used for analysis of ELISA data, and unpaired t-tests with Welch's correction were used for analysis of body, ovary and uterus weights at 16 weeks of age. Significance was set at p < 0.05.

## Results

### Disrupted estrous cycling in prenatally androgenized rats

Compared with the regular 4–5 day estrous cycles for control rats 15–16 weeks of age, prenatally androgenized rats exhibited longer and irregular estrous cycles.

### Prenatal exposure to testosterone results in elevated testosterone and androstenedione in young adult but not early adolescent female rats

**Fig 1:**
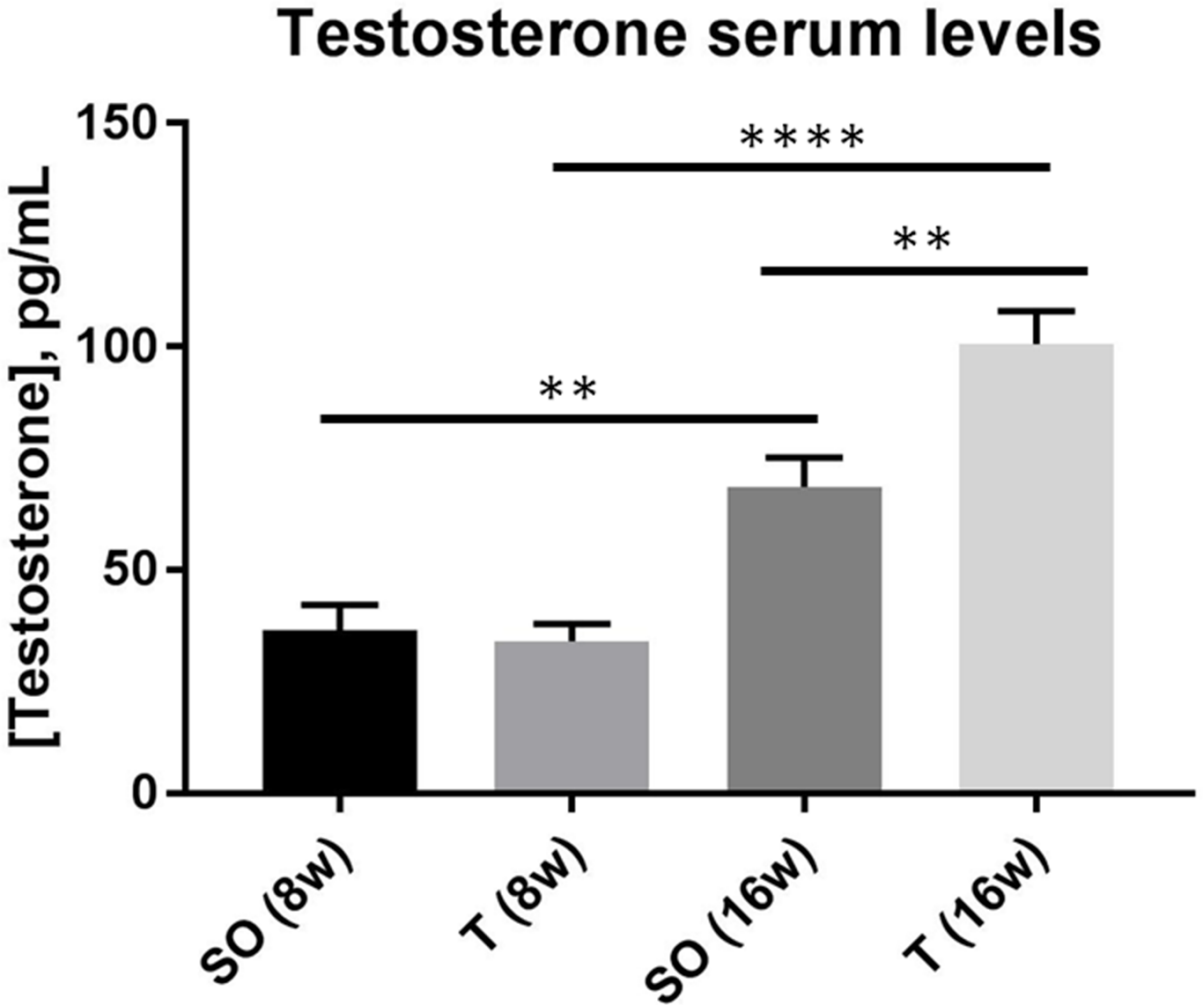
Serum testosterone concentration is significantly higher in adult, but not early adolescent, prenatally androgenized rats compared with controls. Sprague-Dawley rats were prenatally exposed to sesame oil or 5mg testosterone each day during gestation days 16–19. Serum samples, collected by tail vein blood draw at 8 and 16 weeks of age, were assayed by ELISA for T. No significant difference in [T] was detected at 8 weeks of age, but rats prenatally exposed to testosterone exhibited significantly greater [T] as young adults compared to rats exposed to sesame oil, with a 30% greater level in the PNA rats (16 weeks of age, n = 8 for each group, 2-way ANOVA with Tukey’s, ** p < 0.01, **** p < 0.0001). Values are means ± SEM. SO, sesame oil prenatal exposure; T, testosterone prenatal exposure.

**Fig 2:**
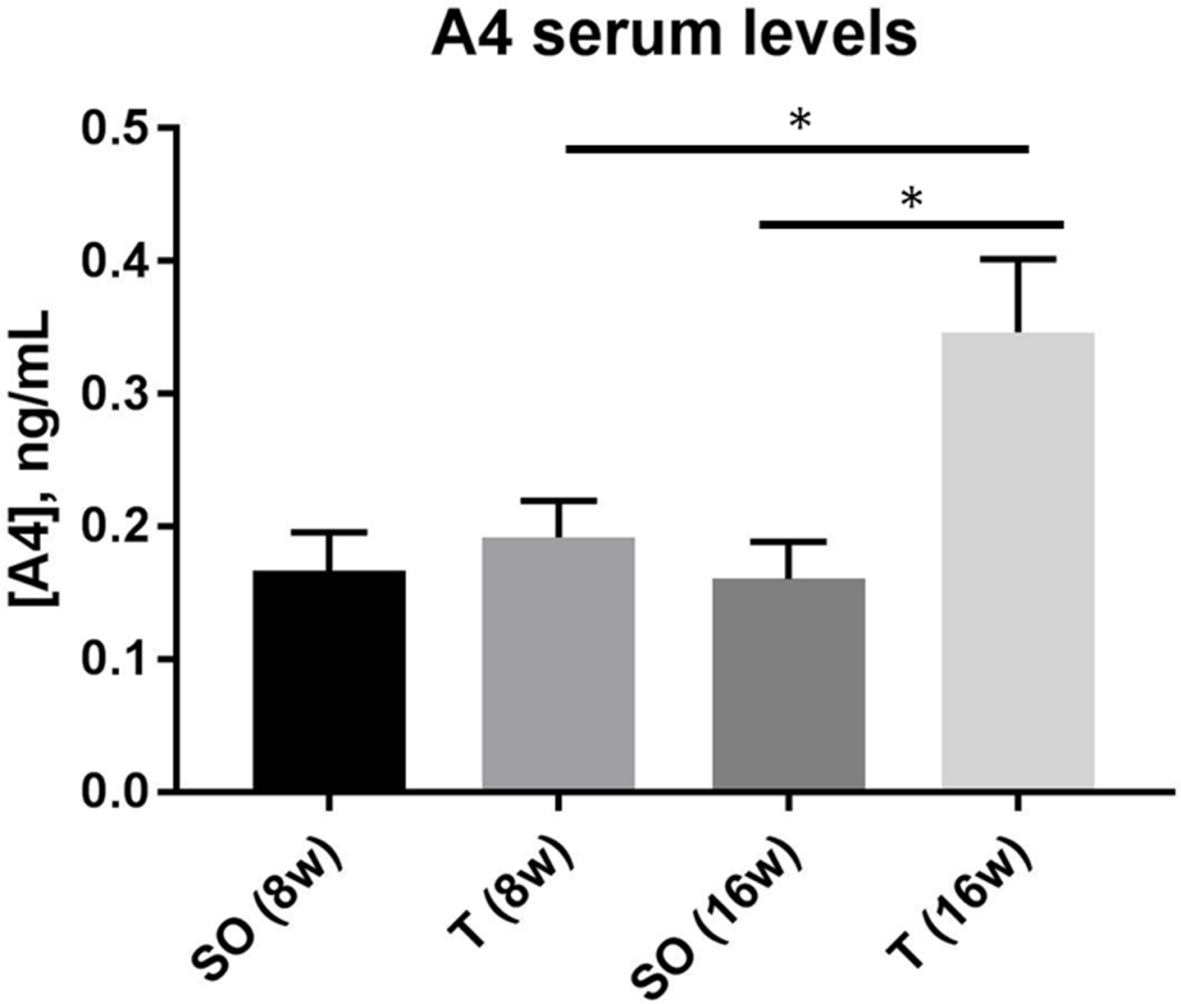
Serum A4 concentration is significantly higher in adult, but not early adolescent, prenatally androgenized rats. Sprague-Dawley rats were prenatally exposed to sesame oil or 5mg testosterone each day during gestation days 16–19. Serum samples, collected by tail vein blood draw at 8 and 16 weeks of age, were assayed by ELISA for A4. No significant difference in [A4] was detected at 8 weeks of age, but rats prenatally exposed to testosterone exhibited significantly greater [A4] as young adults compared to rats exposed to sesame oil, with a two-fold greater level in the PNA rats (16 weeks of age, n = 8 for each group, 2-way ANOVA with Tukey’s, * p < 0.05). Values are means ± SEM. SO, sesame oil prenatal exposure; T, testosterone prenatal exposure.

Rats exposed to 5 mg testosterone in utero during days 16–19 of gestation demonstrated no significant difference in either of these androgens at 8 weeks of age compared to rats exposed only to vehicle (Figs 1 and 2). There was a significant increase in both testosterone and androstenedione from 8 to 16 weeks of age in the PNA rats. Control rats also exhibited a significant increase in testosterone, but not androstenedione, from 8 weeks of age to 16 weeks of age. Comparison of PNA rats with controls indicated that there was significantly elevated serum testosterone (Fig 1) and serum androstenedione (Fig 2) levels in PNA rats at 16 weeks of age compared with rats exposed only to vehicle. The mean serum testosterone level in the prenatally androgenized rats (100.6 pg/mL ± 7.4) was approximately 40% greater than the level in control rats at 16 weeks (68.6 pg/mL ± 6.6; p = 0.0080). The mean serum androstenedione level in the prenatally androgenized rats (0.35 ng/mL ± 0.055) was more than twice the level in control rats at 16 weeks (0.16 ng/mL ± 0.028; p = 0.0123).

### No change in ovary, uterine, or total body weight

There was no significant difference in the body weights at 16 weeks of age in PNA rats compared with controls. Ovaries and uterus were carefully dissected from each animal following perfusion. The mean absolute ovarian weight was approximately 13% lower in prenatally androgenized rats (Table 1, p = 0.0090); however, the ovary weight relative to total body weight was not significantly different. Ovaries from the PNA rats exhibited polycystic morphology (S1 Fig). No significant difference in the absolute uterine weight was found in the PNA rats compared to the control rats. Other differences observed in the reproductive anatomy were an increase in anogenital distance in some of the female PNA rats and vaginal fusion in approximately half of the PNA rats (S2 Fig).

**Table 1:** Total body weight and reproductive organ weight.

	Control	PNA	P value
Body weight at 16 weeks of age (g)	297.4 ± 4.1	290.0 ± 10.9	0.5377
Ovary weights (g)	0.07151 ± 0.0025	0.06201 ± 0.0025 **	0.0090
Relative ovary wt (% body wt)	0.0238 ± 0.0008	0.02156 ± 0.001	0.0839
Uterine weight (g)	6.253 ± 0.439	6.326 ± 0.379	0.9002
Relative uterus wt (% body wt)	2.075 ± 0.137	2.206 ± 0.1284	0.4910

Sprague-Dawley rats prenatally exposed to sesame oil or testosterone were weighed at 16 weeks of age, then anesthetized, perfused with normal saline, and the reproductive organs carefully dissected and weighed. PNA rats exhibited significantly lower absolute ovary weights compared to controls (~13% decrease, n = 17 and n = 16 for controls and PNA, respectively), although the relative ovary weight is not significantly different. Values are means ± SEM. There was no significant difference in body weight between the two groups.

Two-tailed *t*-tests with Welch’s correction,

** p < 0.01.

### Prenatally androgenized rats exhibit significantly higher blood glucose levels both in early adolescence and as young adults

**Fig 3:**
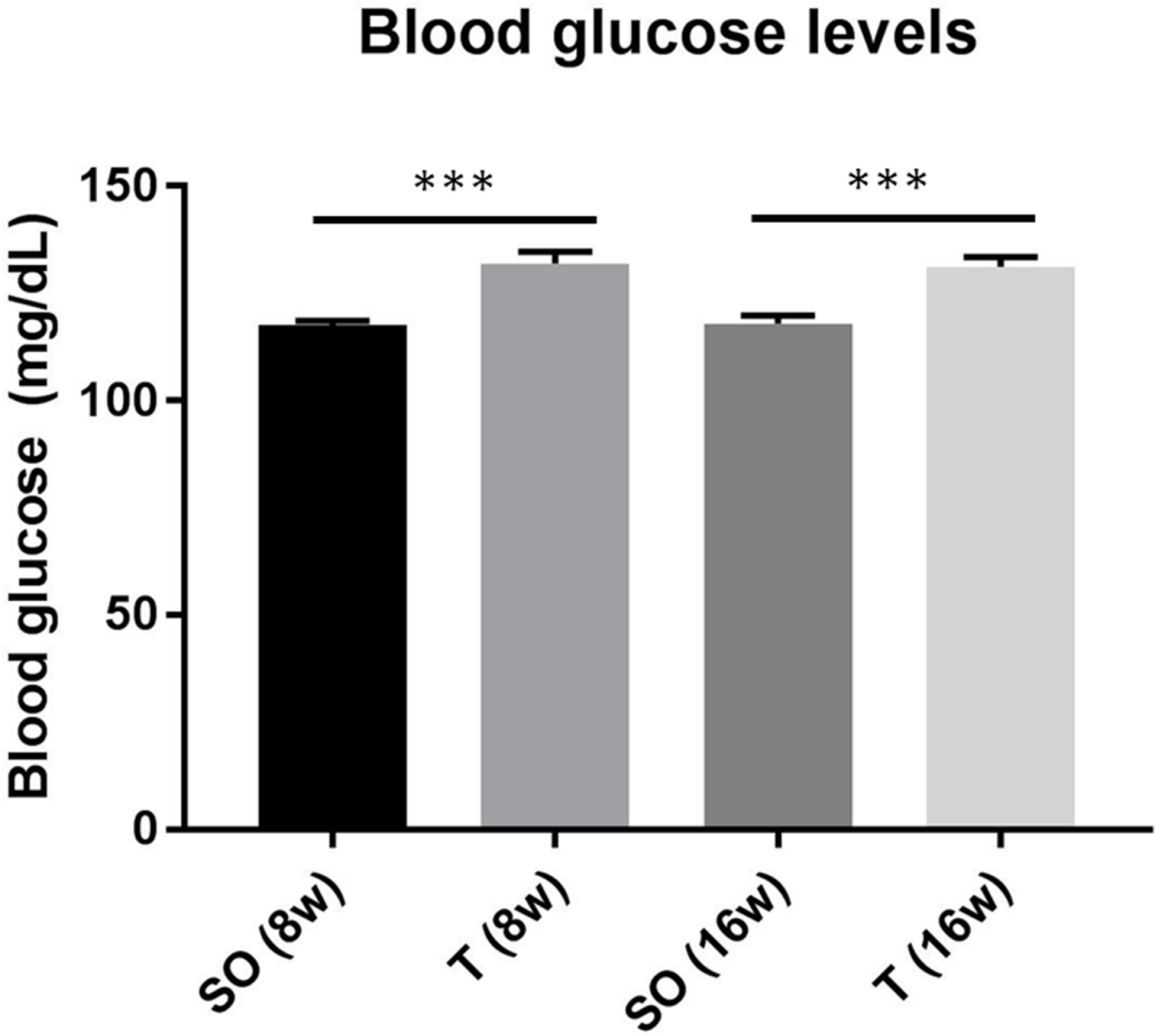
Blood glucose concentration is significantly higher in early adolescent and adult prenatally androgenized rats. Sprague-Dawley rats were prenatally exposed to sesame oil or 5mg testosterone each day during gestation days 16–19. Tail vein blood was used to determine blood glucose using a handheld glucometer. Rats prenatally exposed to testosterone exhibited significantly greater blood glucose (n = 8 for each group, 2-way ANOVA with Tukey’s, *** p < 0.001). Values are means ± SEM. SO, sesame oil prenatal exposure; T, testosterone prenatal exposure.

Tail vein blood sampling at 8 and 16 weeks of age indicated that PNA rats have significantly higher blood glucose levels at both ages. The average blood glucose levels for the control and PNA rats, respectively, were 117.5 and 131.9 mg/dL at 8 weeks of age, and 117.9 and 131.2 mg/dL at 16 weeks of age (Fig 3, *** p < 0.001).

## Discussion

Animal models for PCOS are useful in providing valuable means with which to study the mechanisms underlying the reproductive, endocrine, and metabolic aspects of the disorder, as well as to test novel therapeutic interventions. Rodent models in particular represent an effective and practical way to study PCOS, as they are well-characterized, have a stable genetic background, have short gestation periods and reach adulthood within a few months, are inexpensive, and have been shown to recapitulate the reproductive, endocrine, and metabolic disturbances associated with human PCOS [24, 25, 28–36, 55–59]. While the use of testosterone propionate (TP) to prenatally androgenize rats has been reported to produce limited PCOS-like features in animals [58], PNA rat models using T or DHT exposure between days 16–19 of gestation exhibit anovulation and irregular estrous cycles, elevated LH and increased frequency of LH secretion, polycystic ovaries, greater number of preantral and antral follicles, fewer preovulatory follicles and corpora lutea, elevated T and estradiol, dyslipidemia, greater body weight, and insulin resistance [29, 30, 34, 35, 55, 58–60]. For this reason, we chose a PNA rat model using T exposure between gestation days 16–19, which mimics the endogenous T surge in male rats [61], to analyze A4 levels at early adolescence and young adulthood.

Female laboratory rats are reported to reach sexual maturity, defined as vaginal opening at 6 weeks of age by some reports [62] and at an average age of 50 days by others [63]. In our rats, ~50% have patent vaginas at 6 weeks of age, and 100% at 7.5 weeks of age. This milestone marks the beginning of adolescence. We examined our rats at 8 weeks of age, at which time we were able to determine estrous phase over the course of a week for all rats which did not have vaginal fusion. Vaginal cytology at 8 weeks of age indicated regular 4–5 day estrous cycles for most of the control rats, with all exhibiting regular estrous cycles by 15 weeks of age. During the course of our study, we also observed polycystic ovary morphology, increased anogenital distance, and vaginal fusion in some of the PNA rats, similar to what others have reported in rats prenatally androgenized with DHT or T [58].

In this model for PCOS, we found that there was no significant difference in either T or A4 at 8 weeks of age when comparing control and PNA rats. In both the control and the PNA rats, T was significantly higher at 16 weeks of age compared with 8 weeks of age, possibly reflecting a rise in this hormone that corresponds with continued sexual maturation and development. No significant difference in A4 was observed in the control rats between 8 and 16 weeks of age. Interestingly, both serum T and A4 were significantly higher in the PNA rats compared to controls at 16 weeks of age. This suggests that, in this model, hyperandrogenemia is not yet established at early adolescence but rather develops between early adolescence and early adulthood in the PNA rats.

In humans, the relative effectiveness of using standard diagnostic criteria for identifying adolescents with PCOS is not well established, including the use of biochemical hyperandrogenemia [3]. Greater levels of hyperandrogenism have been noted in adolescent females that are overweight and obese compared to normal weight females [64], suggesting that obesity can exacerbate androgen levels in adolescents. However, it is also possible that elevated androgens may contribute to susceptibility to weight gain in these individuals, which would underscore the importance of identifying adolescents with hyperandrogenemia so as to initiate preventative care to maintain metabolic health and minimize the more severe PCOS sequelae. In our study, it is worth noting that the PNA rats did not have a significantly greater body weight than controls between 3 and 16 weeks (Table 1), indicating that hyperandrogenemia presents before obesity is apparent in this model, and lending support to the hypothesis that hyperandrogenemia promotes weight gain. Further research is needed to determine if the body composition (i.e., relative proportion of adipose tissue) differs in the young adult PNA rats compared to controls, despite no significant difference in total body weight. Interestingly, blood glucose levels were significantly higher in the PNA rats than the control rats at both 8 and 16 weeks of age, suggesting that this aspect of metabolic dysfunction has already developed by early adolescence and prior to significant changes in serum A4 levels or total body weight. Together, these findings indicate that there is utility in assessing serum androgen levels prior to early adulthood and the development of certain aspects of metabolic dysfunction (e.g., obesity), but that there may be some evidence of metabolic dysfunction (e.g., glucose intolerance) that develops prior to apparent hyperandrogenemia.

Women with PCOS typically have hyperandrogenism, which may be identified by clinical presentation or through biochemical analysis of serum androgens. Free or total T is often used to support a diagnosis of PCOS, although A4 has also recently received attention for its utility in identifying hyperandrogenemic women with PCOS [18, 37, 45, 47]. In a study involving women meeting the Rotterdam criteria for PCOS, most exhibited both elevated serum testosterone and A4; notably, there was also a sizeable subset of women who exhibited elevated serum A4 but had normal testosterone levels [47]. Georgopoulos and colleagues reported that elevated A4 (>3.8 ng/mL) in women with PCOS is associated with a more severe phenotype, including elevated LH, LH:FSH ratio, T, DHEA-S, and free androgen index (FAI), as well as increased ovarian volume and greater mean number of follicles in hyperandrogenic women with PCOS [37]. Others have found that, along with calculated fT and FAI, A4 best predicted PCOS in women between the ages of 18 and over 50 [45]. A significant correlation between A4 and both T and FAI has been confirmed by another group, who also found that up to 90% of women with PCOS may be hyperandrogenic, including some women who were initially characterized as having the oligo-anovulation and PCO morphology phenotype [18]. Together, these findings underscore the importance of measuring both testosterone and A4 when evaluating a patient for PCOS.

Though multiple studies demonstrate A4 elevation in women with PCOS, the biological significance of this elevation is debated. A4 is considered a weak androgen, produced as an intermediate in the synthesis of T. As elevated A4 is associated with a more severe PCOS phenotype, it may contribute to the worsening of endocrine and reproductive parameters associated with PCOS [37]. Some have shown that the elevation in A4 strongly and negatively associates with insulin sensitivity, a relationship that was not observed for insulin sensitivity with T, DHEA, or DHEA-S. This suggests that measurement of A4 may be useful in predicting metabolic risk in women with PCOS [47]. However, others have reported that PCOS women with elevated A4 but normal free testosterone have no significantly increased risk of metabolic parameters compared to PCOS women with normal levels of both A4 and T, and that a higher A4/free T ratio may even be associated with a beneficial metabolic profile [48]. In the latter study, the median BMIs of both control and PCOS women were lower than those for control and PCOS women in the study by O'Reilly and colleagues, which may partially account for this discrepancy. Women in the latter study also had a lower median FAI and higher sex hormone binding globulin (SHBG) levels for both controls and PCOS, indicating a less severe endocrine presentation compared with those in the O'Reilly study. However, it is also possible that greater levels of A4 represent greater potential for conversion to T, which itself contributes to a more severe phenotype. In a study of women with biochemical hyperandrogenemia, obese women were found to have lower A4 and an increased T:A4 ratio, suggesting that obesity may allow further conversion of A4 to T [65], which could in turn worsen metabolic dysfunction. Although the relationship between androgens and obesity is likely complex and multi-factorial, these data support a positive relationship between BMI and androgen levels, both of which can contribute to metabolic risk in women with PCOS.

## Conclusions

In conclusion, our results indicate that a rat PNA model for PCOS exhibits elevations in both T and A4 in young adult females, similar to what has been reported for adult women with PCOS and further validating the utility of this model. Moreover, elevation in each of these androgens occurs sometime between early adolescence and adulthood in this rodent model, and precedes the appearance of significant weight gain, suggesting that elevated T and A4 may play a role in the development of obesity in this model for PCOS. Further investigation is needed to determine if A4 itself plays a biological role in the development of obesity or metabolic dysfunction, independent from T.

## Supporting information

S1 Fig Prenatally androgenized rats exhibit polycystic ovary morphology. Spraque-Dawley rats were prenatally exposed to sesame oil or 5 mg testosterone each day during gestation days 16–19. Compared with ovaries from control animals (left image), ovaries from prenatally androgenized rats (right image) exhibited more cystic, preantral, and antral follicles, with fewer corpora lutea.
(TIF)

S2 Fig Some prenatally androgenized rats exhibit vaginal fusion. Sprague-Dawley rats were prenatally exposed to sesame oil or 5 mg testosterone each day during gestation days 16–19. (A) None of the control rats exhibited vaginal fusion, but just under half of the prenatally androgenized rats exhibited vaginal fusion at 16 weeks of age. (B) Representative images showing a control rat (left) and PNA rat (right).
(TIF)

## Abbreviations

A4: androstenedione
DHEA and DHEAS: dehydroepiandrosterone and dehydroepiandrosterone sulfate
DHT: dihydrotestosterone
ELISA: enzyme-linked immunosorbent assay
FAI: free androgen index
FSH: follicle-stimulating hormone
fT: free testosterone
LH: luteinizing hormone
PBS: phosphate-buffered saline
PCOS: polycystic ovary syndrome
PNA: prenatally androgenized
SO: sesame oil
T: testosterone
