# Supplementary figures and images for: Elevated androstenedione in young adult but not early adolescent prenatally androgenized female rats
Source: PLoS One. 2018 May 3;13(5):e0196862. doi: 10.1371/journal.pone.0196862 (PMC5933698; doi:10.1371/journal.pone.0196862)

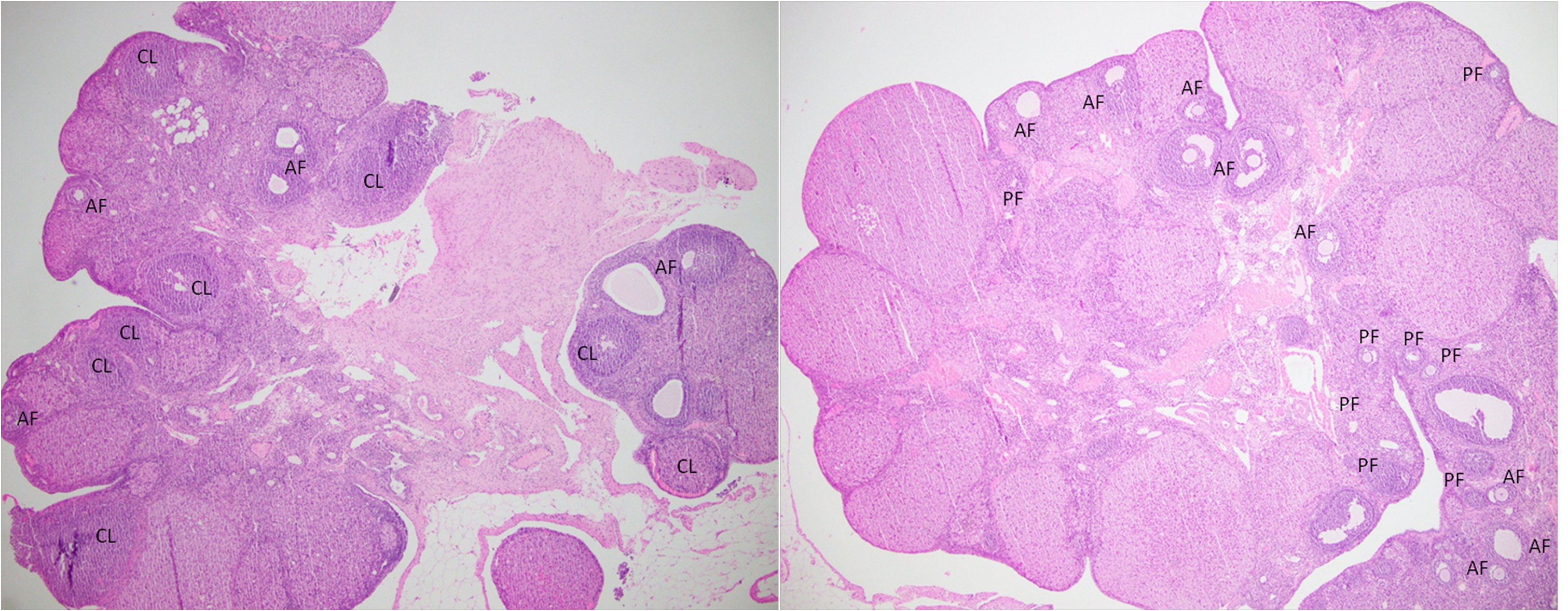

Supplement: S1 Fig — Spraque-Dawley rats were prenatally exposed to sesame oil or 5 mg testosterone each day during gestation days 16–19. Compared with ovaries from control animals (left image), ovaries from prenatally androgenized rats (right image) exhibited more cystic, preantral, and antral follicles, with fewer corpora lutea. (TIF) [file pone.0196862.s001.tif]

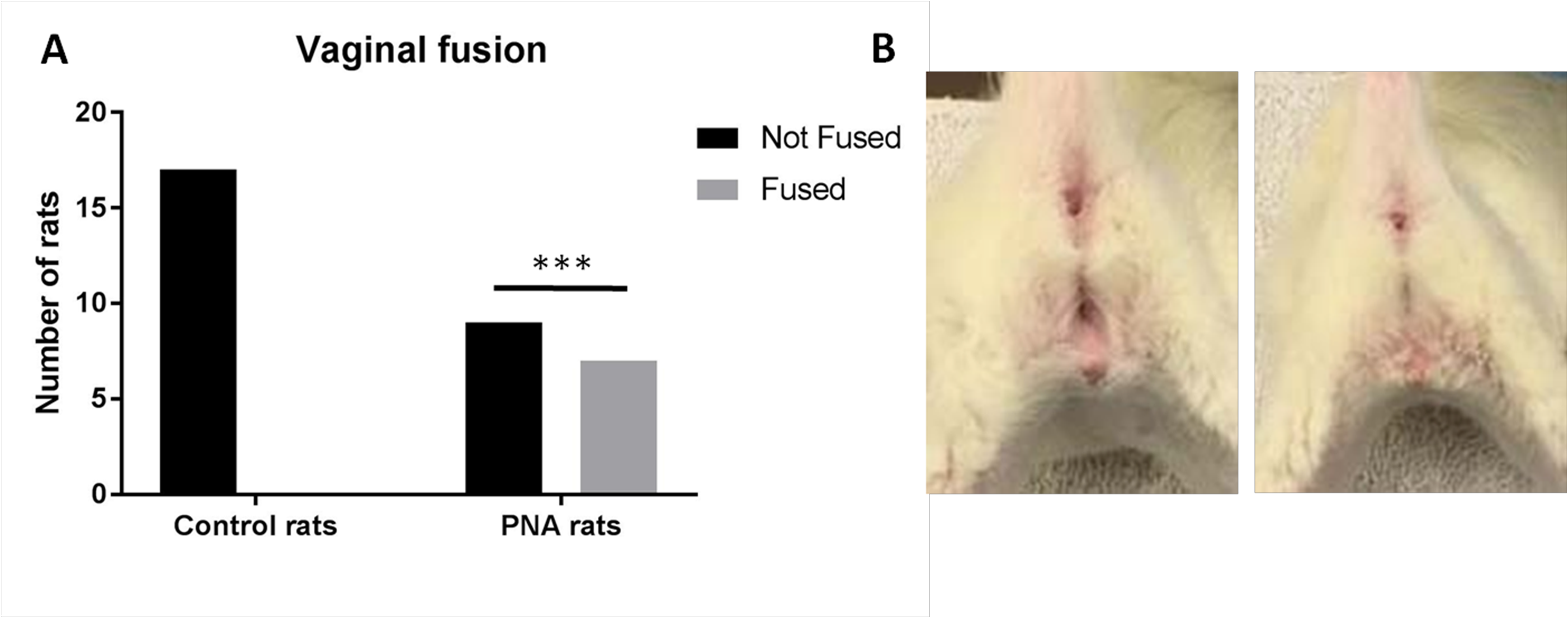

Supplement: S2 Fig — Sprague-Dawley rats were prenatally exposed to sesame oil or 5 mg testosterone each day during gestation days 16–19. (A) None of the control rats exhibited vaginal fusion, but just under half of the prenatally androgenized rats exhibited vaginal fusion at 16 weeks of age. (B) Representative images showing a control rat (left) and PNA rat (right). (TIF) [file pone.0196862.s002.tif]
